# Supplementary material for: Fabrication of triboelectric polymer films via repeated rheological forging for ultrahigh surface charge density
Source: Nat Commun. 2022 Jul 14;13:4083. doi: 10.1038/s41467-022-31822-2 (PMC9283396; doi:10.1038/s41467-022-31822-2)
Supplement: Supplementary file 1 — Supplementary Information [file 41467_2022_31822_MOESM1_ESM.pdf]

Supplementary Information for

Fabrication of triboelectric polymer films via repeated rheological forging for ultrahigh surface charge density

Zhaoqi Liu[1,2,3 ‡], Yunzhi Huang[2 ‡], Yuxiang Shi[1,3], Xinling Tao[1,3], Hezhi He[2], Zhao-Xia Huang[2]*, Zhong Lin Wang[1,3,4], Xiangyu Chen[1,3]*, Jin-Ping Qu[2,5]*

[1]CAS Center for Excellence in Nanoscience, Beijing Institute of Nanoenergy and Nanosystems, Chinese Academy of Sciences, 100083 Beijing, China.

[2]National Engineering Research Center of Novel Equipment for Polymer Processing; Key Laboratory of Polymer Processing Engineering, Ministry of Education; Guangdong Provincial Key Laboratory of Technique and Equipment for Macromolecular Advanced Manufacturing; Department of Mechanical and Automotive Engineering, South China University of Technology, 510641 Guangzhou, China

[3]School of Nanoscience and Technology, University of Chinese Academy of Sciences, 100049 Beijing, China.

[4]School of Materials Science and Engineering, Georgia Institute of Technology, Atlanta, GA 30332-0245, USA.

[5]School of Chemistry and Chemical Engineering, Huazhong University of Science & Technology, 430074 Wuhan, China

*Corresponding author: Z.-X. H. (mehuangzx@scut.edu.cn), X. C. (chenxiangyu@binn.cas.cn), J.-P. Q. (jpqu@scut.edu.cn).

‡ These authors contributed equally: Z. L. and Y. H.

This file includes:

Supplementary Figure S1 to S13

Supplementary Table S1 to S4

	RRF-FEP1	RRF-FEP2	RRF-FEP3	RRF-FEP4
t_{on}	0.5s	0.5s	0.5s	0.5s
t_{off}	0.7s	0.95s	2.1s	2.63s
P_{peak}	50MPa	50MPa	50MPa	50MPa
T	300℃	300℃	300℃	300℃

Supplementary Table S1: The detailed parameters of RRF process.

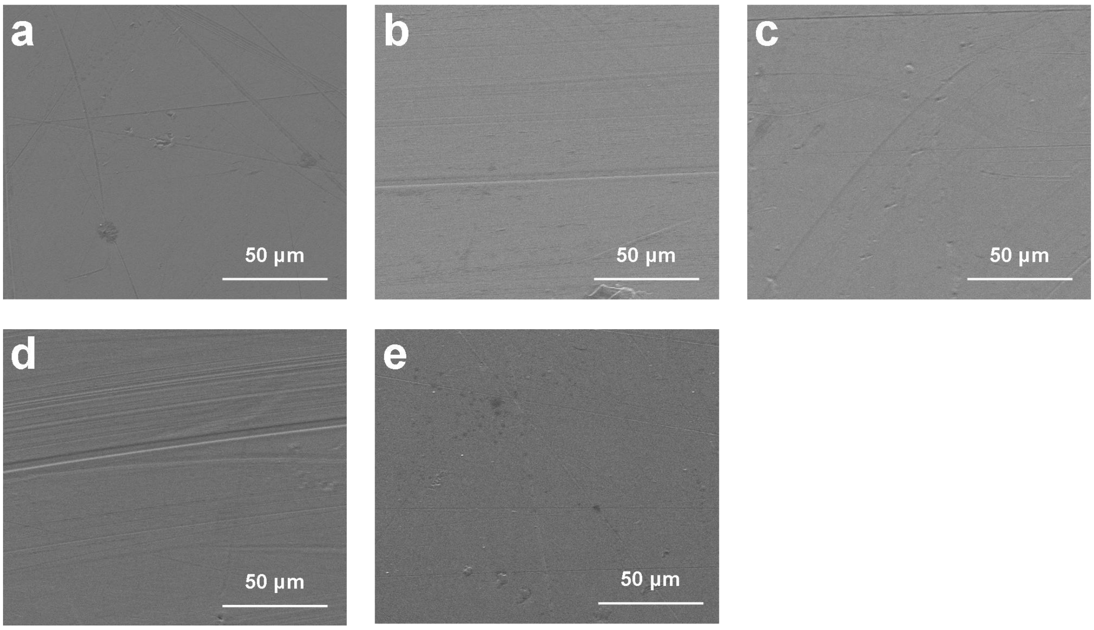

Supplementary Figure S1: The surface morphology of FEP given by SEM.
The surface morphology of a) commercial FEP b) RRF-FEP1 c) RRF-FEP2 d) RRF-FEP3 e) RRF-FEP4.

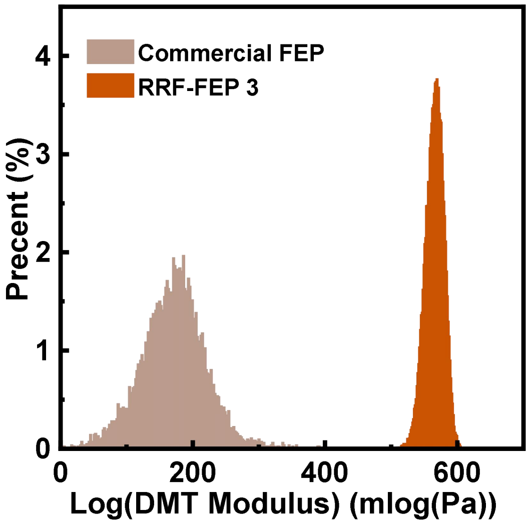

Supplementary Figure S2: The surface elastic modulus of commercial FEP and RRF-FEP3 is measured by AFM.

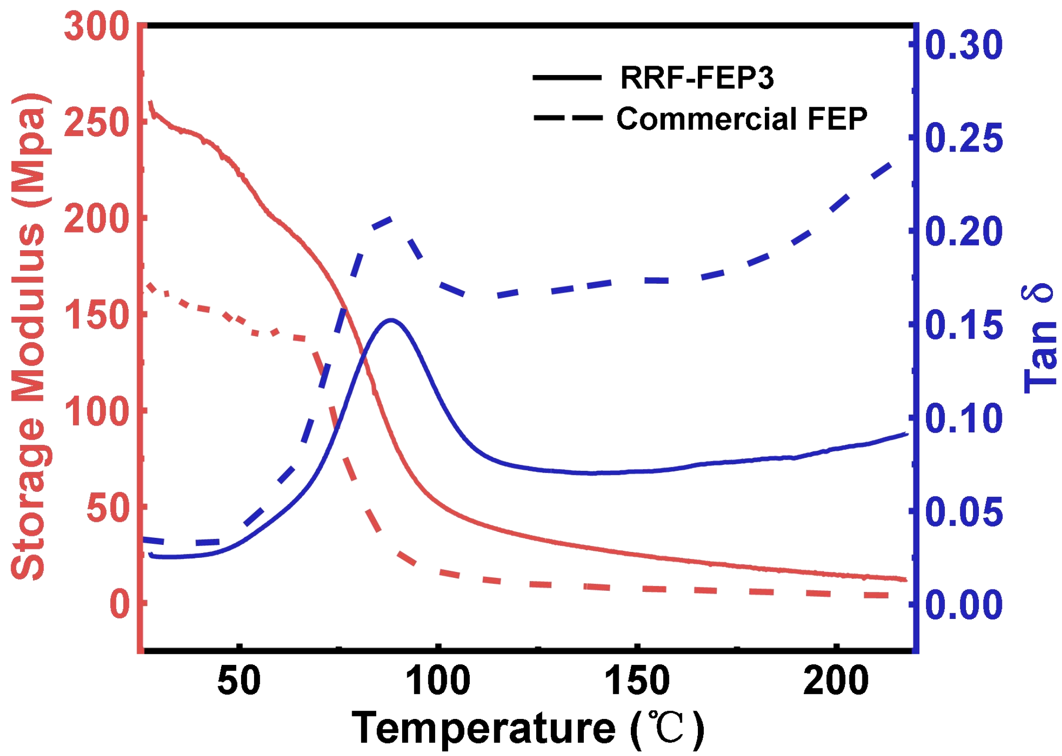

Supplementary Figure S3: The storage modulus and shear angle of commercial FEP and RRF-FEP3 measured by DMA.

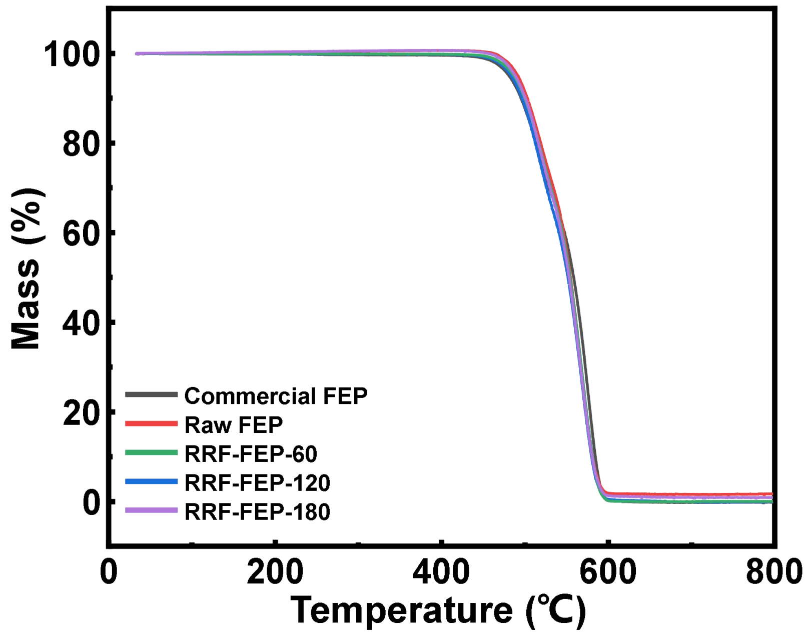

Supplementary Figure S4: Thermogravimetric analysis (TGA) diagram of commercial FEP and RRF-FEP3 with different RRF cycles.

Reference	FEP thickness (μm)	σ_{stable} (μC·m-2)	Method of material	Reference
1	50	81.25	original FEP	[29]
2	50	120.9	original FEP	[19]
3	50	140	original FEP	[18]
4	127	32	surface modify	[16]
5	50	40	surface modify	[17]
6	50	165.6	surface modify	[15]
7	50	110	TENG with special design	[14]
8	30	220	TENG with special design	[31]
9	50	200	ion inject	[5]
10	50	230	ion inject	[21]
11	30	230	ion inject	[20]
12	30	240	ion inject	[22]

Supplementary Table S2: Surface charge density of FEP films in previous studies.

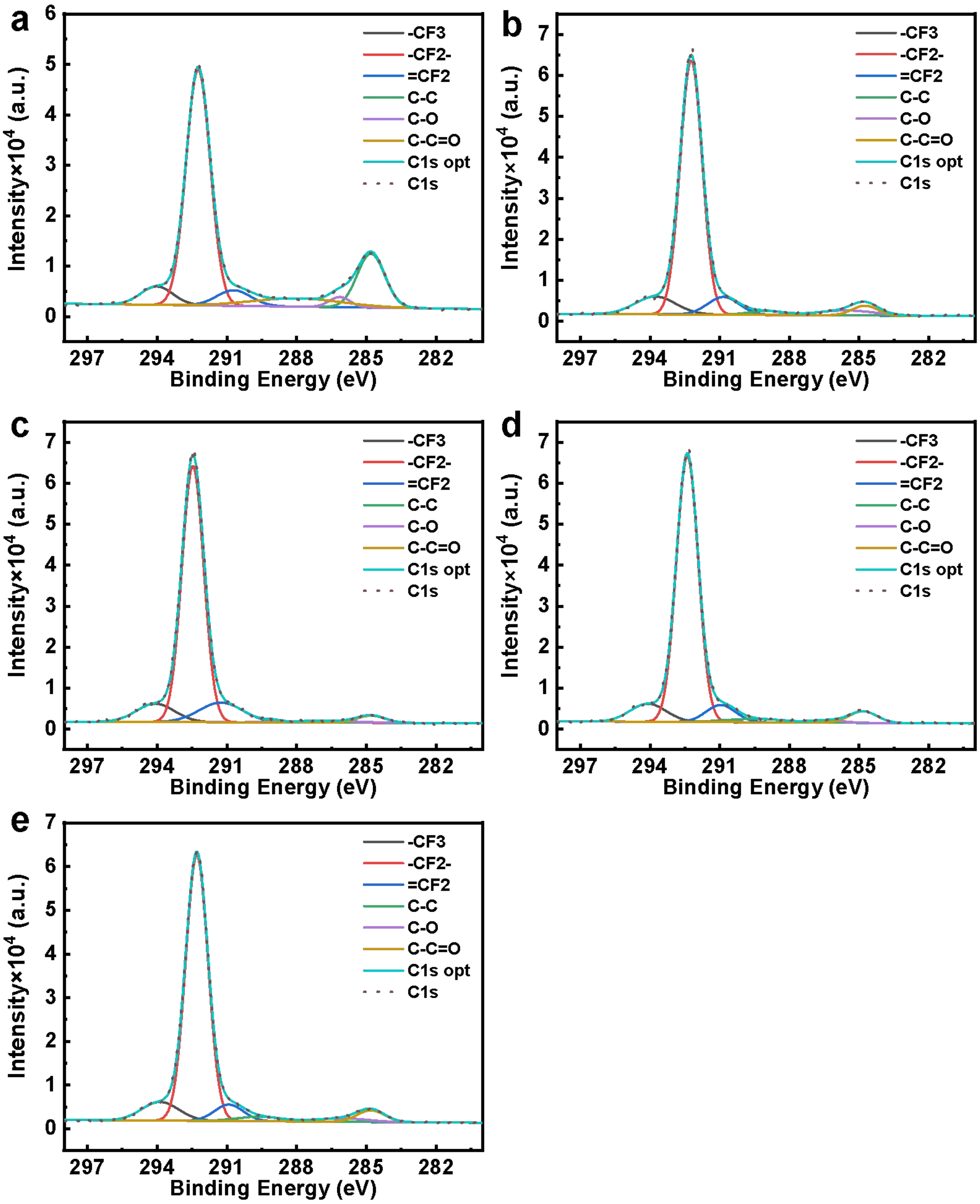

Supplementary Figure S5: The C 1s spectra of FEP given by XPS.

a) commercial FEP b) RRF-FEP1 c) RRF-FEP2 d) RRF-FEP3 e) RRF-FEP4.

	-CF3	-CF2-	=CF2	C-C	C-O	C-C=O
Commercial FEP	6.40%	61.63%	5.74%	16.00%	2.29%	7.94%
RRF-FEP1	9.63%	74.09%	7.41%	1.87%	3.68%	3.32%
RRF-FEP2	9.33%	74.68%	11.44%	0.33%	1.61%	2.61%
RRF-FEP3	8.58%	76.90%	6.64%	2.68%	0.96%	4.24%
RRF-FEP4	8.90%	76.09%	6.08%	2.63%	2.22%	4.08%

Supplementary Table S3: Proportion of combined state of various C1s in FEP measured by XPS.

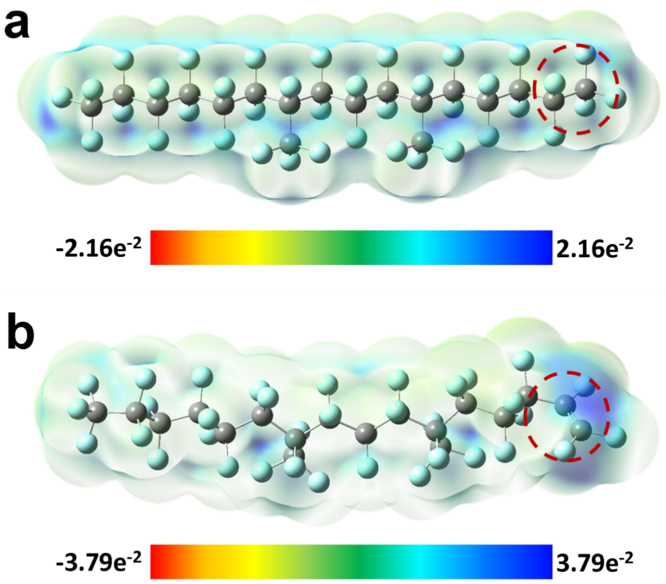

Supplementary Figure S6: Electrostatic potential maps of FEP.

a) no double bond in the chain b) FEP terminated by carbon-carbon double bond.

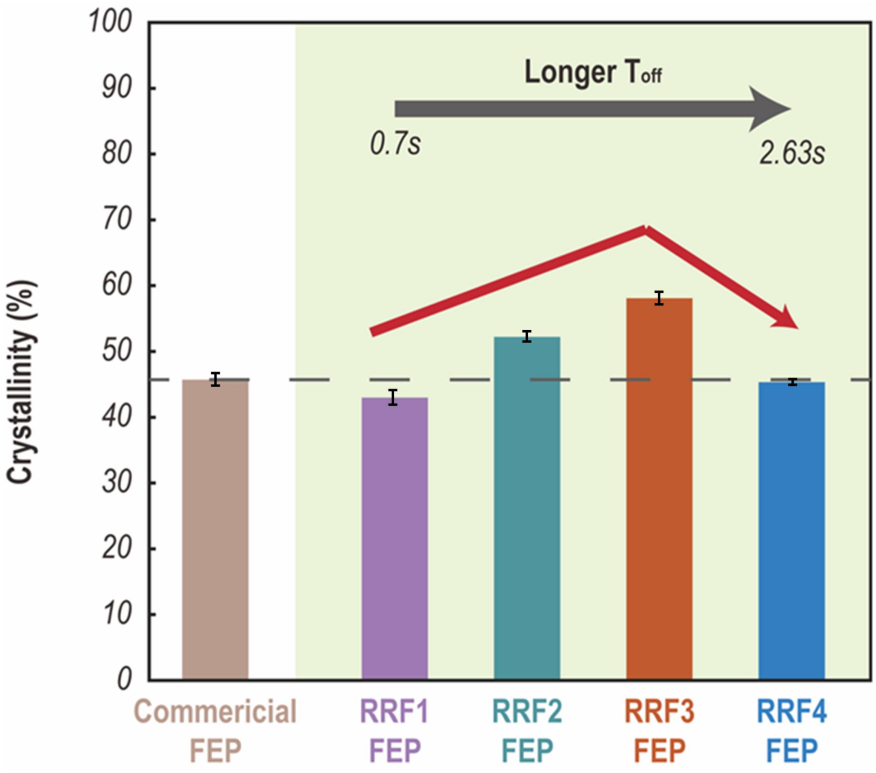

Supplementary Figure S7: The variation trend of crystallinity of RRF-FEPs. The error bar is obtained by the standard deviation of the fitting.

	Lifetimes(ns)		Intensities
**Commercial FEP**	τ_1	0.001	88.037 %
	τ_2	0.1726	11.638 %
	τ_3	2.5307	0.325 %
**RRF-FEP 1**	τ_1	0.001	87.187 %
	τ_2	0.172	12.405 %
	τ_3	2.6546	0.408 %
**RRF- FEP 3**	τ_1	0.001	88.421 %
	τ_2	0.1623	11.132 %
	τ_3	2.5196	0.447 %

Supplementary Table S4: Lifetime and intensity of positron annihilation in FEP measured by PALS.

The semi-empirical formula for calculating the cavity size from the o-Ps lifetime is as follows:

$$\frac{1}{\tau_3} = \lambda_3\,(ns^{-1}) = 2(1 - \frac{R}{R + \Delta R} + \frac{1}{2\pi}\sin\,(\frac{2\pi R}{R + \Delta R}))$$

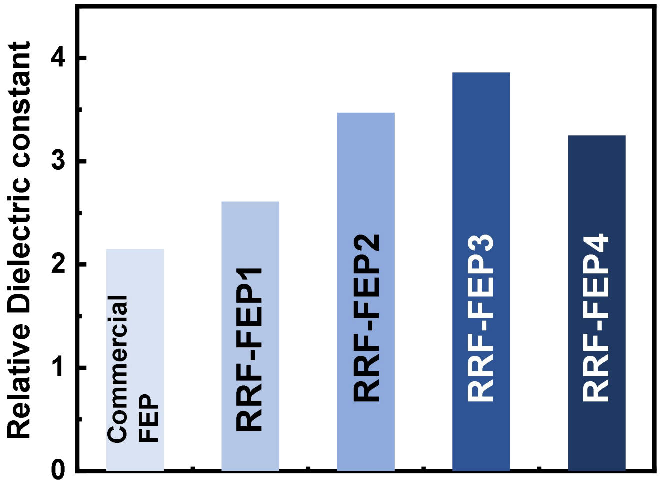

Supplementary Figure S8: The relative dielectric constant of commercial FEP and RRF-FEP.

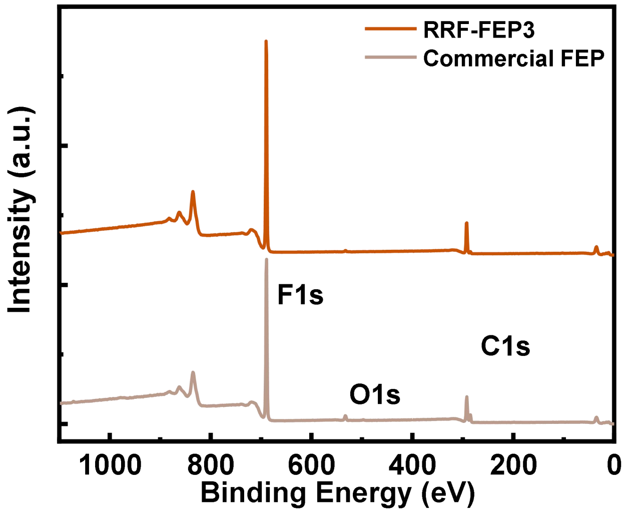

Supplementary Figure S9: XPS spectra of RRF-FEP3 and commercial FEP.

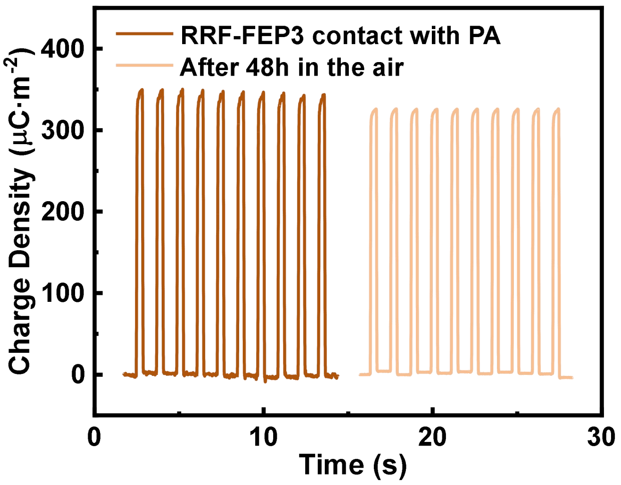

Supplementary Figure S10: Charge density of PA-FEP after corona poling and after placing in the air for 48h.

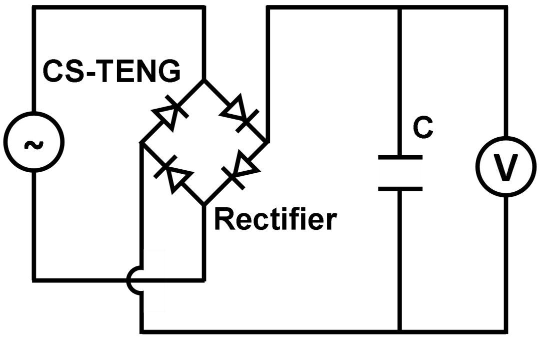

Supplementary Figure S11: Circuit diagram of charging commercial capacitor.

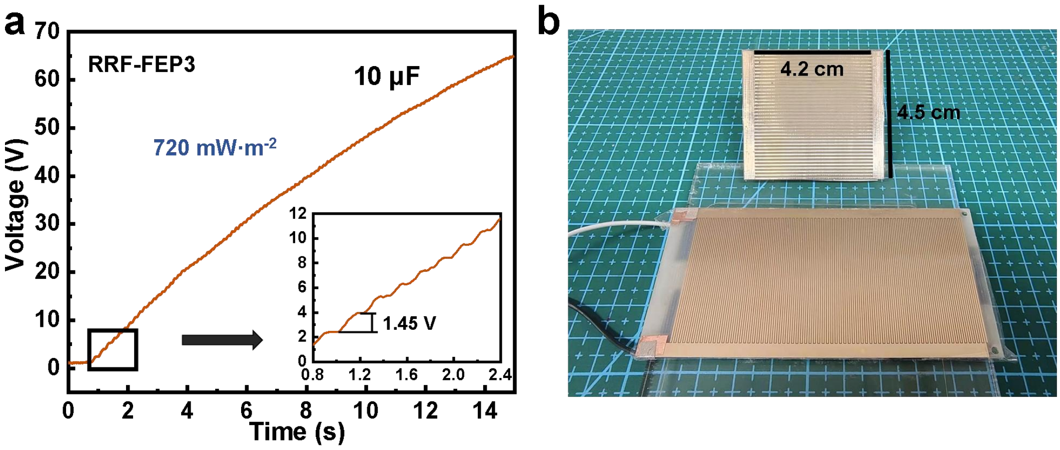

Supplementary Figure S12: FEP charging capacity. a) Charging curve of 10 μF commercial capacitor using the RRF-FEP 3 of grating-structured freestanding TENG. b) The structure of grating-structured freestanding TENG.

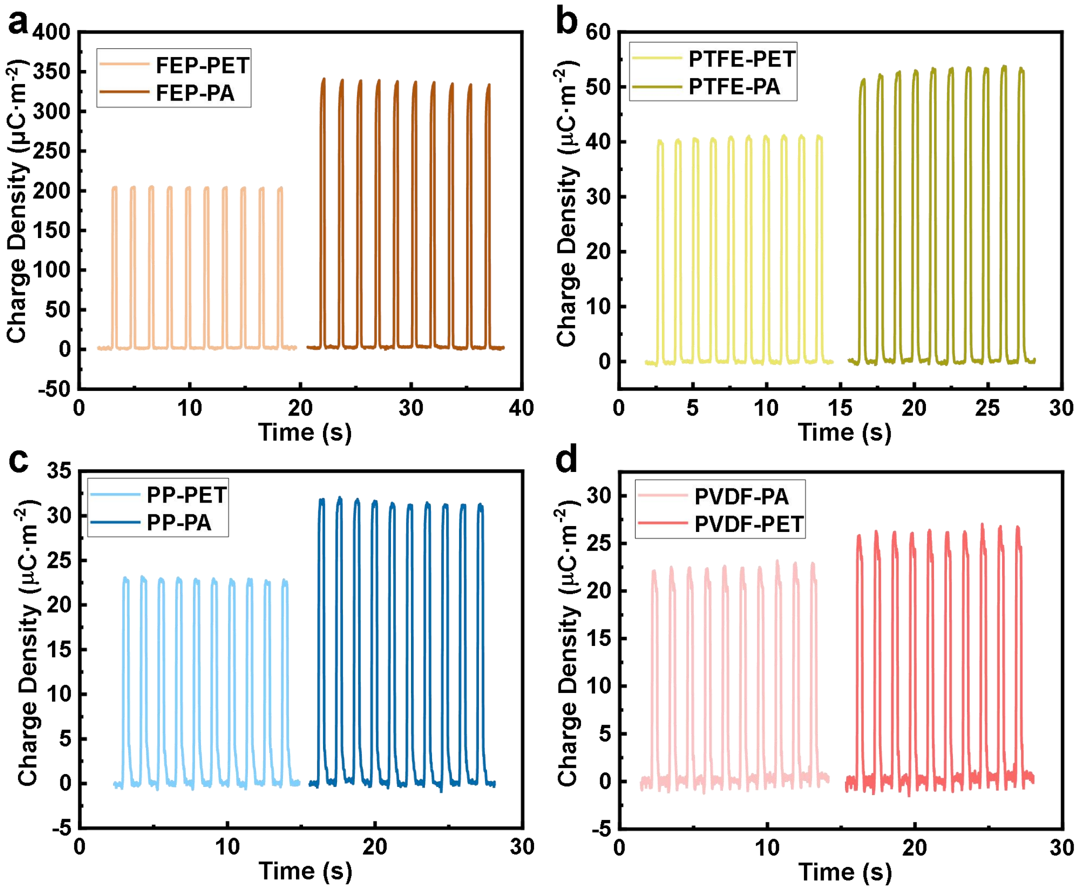

Supplementary Figure S13: Charge density of electret a) fluorinated ethylene propylene (FEP) b) polytetrafluoroethylene (PTFE) c) polypropylene (PP) d) Polyvinylidene fluoride (PVDF) contact with Polyethylene terephthalate (PET) and polyamide (PA) films.
