# Supplementary material for: Fabrication of triboelectric polymer films via repeated rheological forging for ultrahigh surface charge density
Source: Nat Commun. 2022 Jul 14;13:4083. doi: 10.1038/s41467-022-31822-2 (PMC9283396; doi:10.1038/s41467-022-31822-2)
Supplement: Supplementary file 3 — Description of Additional Supplementary Files [file 41467_2022_31822_MOESM3_ESM.pdf]

Description of Additional Supplementary Files

File Name: Supplementary Movie 1
Description: Demonstration of repeated rheological forging molding process.

File Name: Supplementary Movie 2
Description: Comparison of charging capacity of commercial FEP and RRF-FEP3.
